# A framework for quantifying the relationship between intensity and severity of impact of disturbance across types of events and species

**DOI:** 10.1038/s41598-017-19048-5

**Published:** 2018-01-15

**Authors:** Aiko Iwasaki, Takashi Noda

**Affiliations:** 1 Graduate School of Environmental Science, Hokkaido University, N10W5, Kita-ku, Sapporo, Hokkaido 060-0810 Japan; 2 Faculty of Environmental Science, Hokkaido University, N10W5, Kita-ku, Sapporo, Hokkaido 060-0810 Japan

## Abstract

Understanding the impacts of natural disturbances on wildlife populations is a central task for ecologists; in general, the severity of impact of a disturbance (e.g., the resulting degree of population decline) is likely to depend primarily on the disturbance intensity (i.e., strength of forcing), type of disturbance, and species vulnerability. However, differences among disturbance events in the physical units of forcing and interspecific differences in the temporal variability of population size under normal (non-disturbance) conditions hinder comprehensive analysis of disturbance severity. Here, we propose new measures of disturbance intensity and severity, both represented by the return periods. We use a meta-analysis to describe the severity–intensity relationship across various disturbance types and species. The severity and the range of its 95% confidential interval increased exponentially with increasing intensity. This nonlinear relationship suggests that physically intense events may have a catastrophic impact, but their severity cannot be extrapolated from the severity–intensity relationship for weak, frequent disturbance events. The framework we propose may help to clarify the influence of event types and species traits on the severity–intensity relationship, as well as to improve our ability to predict the ecological consequences of various disturbance events of unexperienced intensity.

## Introduction

Understanding the impacts of natural disturbances, such as hurricanes, droughts, heat waves, severe cold events, volcanic eruptions, and tsunamis, on wildlife populations is a central task for ecologists^1–5^. In general, the ‘severity’ of impact of a disturbance, often measured as the percentage mortality of a population or kilograms of biomass removed^4,6^, is likely to depend primarily on the ‘intensity’ of disturbance, which we define as the strength of the disturbing force, such as moment magnitude (*Mw*) for an earthquake or wind speed (m/s) for a hurricane^4,6^. In addition, severity can vary with the type of disturbance (e.g., earthquake, hurricane, or heat wave) and the degree of vulnerability of the species^4^. Thus, crucial to understanding severity is how it increases with increasing intensity, and how the relationship varies across different disturbance types or species^1,4,5^.

White and Jentsch^7^ discussed the need to identify the general patterns of severity of natural disturbances across species and event types. Peters *et al*.^8^ proposed a conceptual framework to quantitatively compare disturbance effects across different types of ecosystems and disturbances, and Fey *et al*.^9^ evaluated the temporal pattern of the magnitude of impact (number of deaths) and the frequency of various types of disturbance event for various taxa. However, to our knowledge, no study has quantified both intensity and severity across different types of disturbance and species and evaluated the severity–intensity relationship comprehensively. Such an analysis is hindered by two main obstacles. First, differences in the physical units of the force strength among disturbance events (e.g., wind speed for hurricanes and moment magnitude for earthquakes) make it impossible to directly compare intensity between different disturbance types. Second, interspecific differences in the temporal variability of population size under normal (non-disturbance) conditions make it impossible to use the change in abundance after a disturbance event as a measure of severity: that is, observed population changes might not be caused by the disturbance effect alone, but also by the species-specific natural variability in abundance.

To solve these problems, we devised an analytical method that uses two different dimensionless parameters for ‘return period’ to characterize both the intensity of a disturbance and the severity of its impact. We define the return period for the intensity of a disturbance as the average interval between occurrences of an event with a given force strength in a particular physical unit. In the case of climatic events, the return period is the inverse of the occurrence probability of that event. In the case of rare or occasional events such as tsunamis, the return period is the length of the recording period divided by the number of events of similar magnitude. To calculate a return period for the severity of impacts, we first calculate the effect size of the disturbance on the annual population growth rate, with the annual population growth rate calculated as the logarithm of the ratio of abundance in two consecutive years. This effect size reflects the difference between the annual population growth rate under normal conditions and the rate in the year when the disturbance event occurred. We then calculate the return period as the inverse of the occurrence probability of the effect size calculated above. This return period represents the severity (See Supplementary Fig. S1).

With these new measures, we performed a meta-analysis to examine the severity–intensity relationship of the impact of different types of disturbance on various species (see Supplementary Tables S1 and S2). When examining the causal relationship between intensity and severity, we used only intensity to detect disturbance events^10^. We focused on the severity of impacts of large-scale (≥10 km^2^) disturbance events that had extreme intensity values^11^ and occurred ≤5% of years or less (≥20-year return period). The hypothetical species experienced such events no more than once in two generations (generation time ≤10 years). Large-scale and infrequent disturbances—relative to the body size and generation time of affected species—are ecologically important owing to their large extents and the persistent damage they cause to populations^12^; however, our ecological knowledge of such events is poor because observations of them have been limited^12–15^. In addition, we excluded species with a generation time of less than 1 year, because such species tend to have very high population growth rates and there is a strong likelihood that the growth and reproduction occurring immediately after the disturbance could mask the damage at the first census after the event and thus lead to underestimation of the severity. To assure the accuracy of our estimation of the severity of such events, we carefully selected populations with sufficient quality of abundance data for the analysis (see Methods; Supplementary File S1, section 1; and Supplementary Tables S1 and S3).

Intensity—a measure of the force of disturbances—generally follows either a normal or an extreme temporal distribution^13,15,16^. The return periods of more intense events are represented as becoming longer owing to lower occurrence probabilities in the lower, upper, or both tails of the frequency distribution^15,16^. (For details, see Methods and Supplementary Fig. S1a).

To quantify severity, we assessed the impact as an effect size, which is represented as the change in annual population growth rate between the year when the disturbance event occurred and under normal conditions. The effect size was calculated while accounting for single-subject data (i.e., the event occurred once, so the treatment data show no variability, whereas the control data were gathered over multiple years and do show variability)^17–20^, as follows^17,21,22^:

1 $$E{S}_{i}=\frac{{r}_{i,{\rm{pd}}}-{\bar{r}}_{i,{\rm{n}}}}{SD({r}_{i,{\rm{n}}})},$$

where ${r}_{i,{\rm{pd}}}$ represents the annual population growth rate of species *i* in the year when the disturbance event occurred, ${\bar{r}}_{i,{\rm{n}}}$ represents the mean annual population growth rate of the same species under normal conditions, and $SD({r}_{i,{\rm{n}}})$ denotes the standard deviation of the annual population growth rate under normal conditions for species *i*. The return period of a given severity was calculated as the inverse of the occurrence probability of a given effect size, which was estimated statistically while assuming a normal distribution (For details, see Methods and Supplementary Fig. S1b).

To examine the relationship between intensity and severity, we gathered more than 8000 time series of population abundance from the literature. We then strictly examined whether each dataset met the selection criteria for accurately quantifying the severity of disturbance events. (For details, see Supplementary File S1, section 1; Supplementary Table S3). From this selection, we quantified the intensities of 27 disturbance events representing five types of disturbance and the severities of their effects on the populations of 50 species across a wide range of taxa (eight classes: Mammalia, Aves, Insecta, Crustacea, Bivalvia, Phaeophyceae, Florideophyceae, and Phaeophyceae) with generation times ranging from 1 to 10 years, using data from 17 studies (including three unpublished studies; see Supplementary Tables S1 and S2).

## Results and Discussion

**Figure 1:**
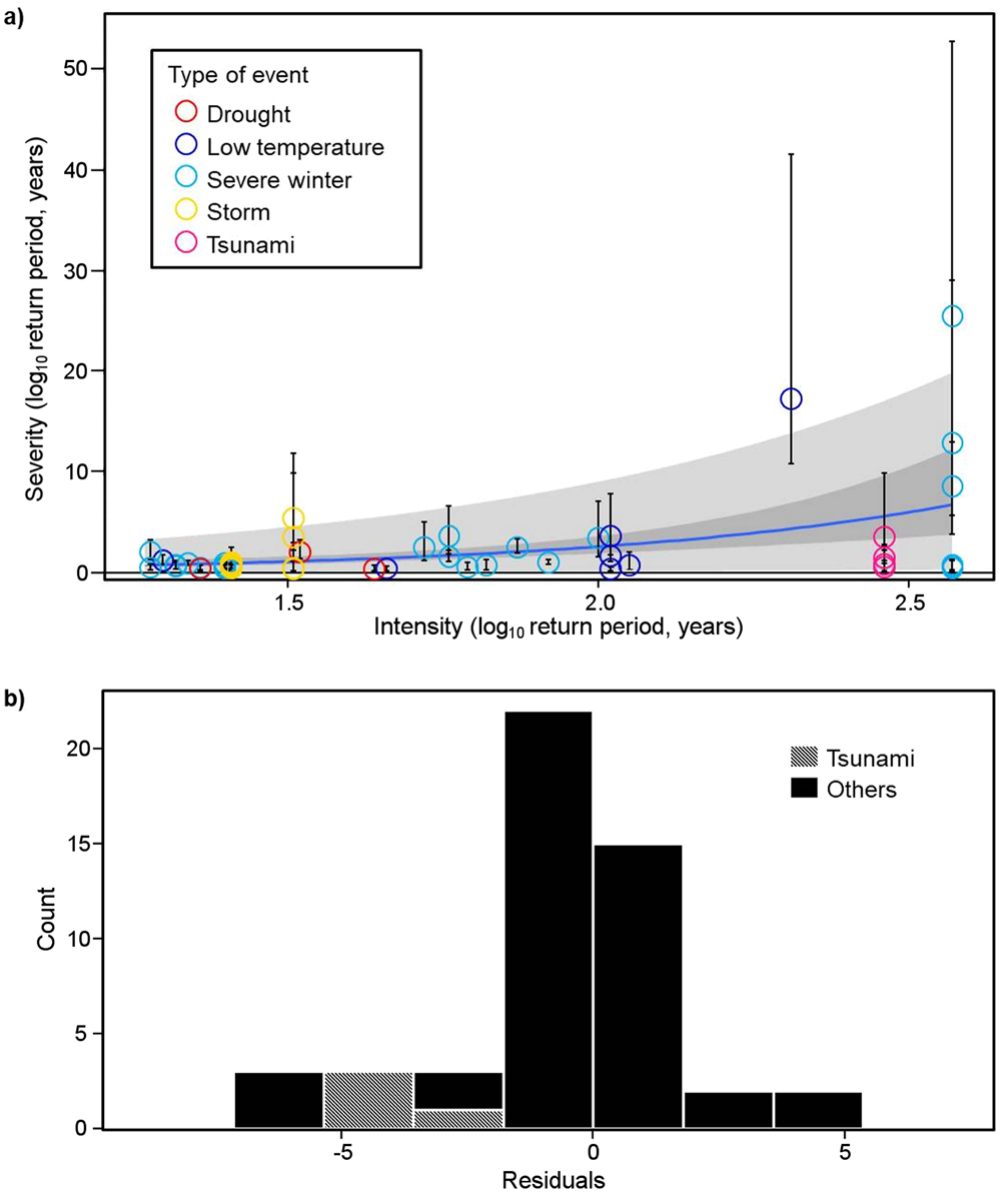
Relationship between severity and intensity. (**a**) The relationship between severity and intensity of 27 disturbance events for five types of disturbance. Each circle is the species-level severity value for a disturbance event (*n* = 50). Vertical lines denote bias-corrected 95% confidence intervals^17^, which were generated by bootstrapping procedures (10 000 iterations)^39^. The blue line represents the estimated mean severity. The darker and lighter shading demarcate the slope of the 95% confidence interval and 95% prediction interval for future observations, respectively. (**b**) Variation in the residuals of species-level severity values from a model describing the relationship between intensity and severity, using a generalized linear model without taking into account the type of disturbance and species. All four severity values (hatched shading) that were detected for tsunamis with return periods of intensity (about 300 years) were relatively small, whereas the two largest residuals of severity were detected for the other two disturbance events with intensity of over 100 years for the return period.

Severity increased exponentially with intensity (Fig. 1a; *P* = 6.35 × 10^−6^): the return period was smaller for severity than for intensity up to an intensity return period of 61 years (log_10_ = 1.8). For disturbances less frequent than that, the return period of severity was greater than that of intensity. Thus, populations could decline more in response to physically intense events occurring less frequently than twice in a century than expected from the relationship for physically weak but frequent events. This result may reflect the fact that a higher frequency of disturbance relative to the life span of an organism must exert strong selection pressure on species to evolve disturbance resistance^1,23–25^.

The severity–intensity relationship (Fig. 1a) also shows that the 95% confidence interval of severity dramatically increases with increasing intensity. In addition, the two largest deviations of severity from the regression curve were for those disturbances with return periods for intensity of >100 years (Fig. 1b). These findings indicate that although intense and rare disturbances could cause catastrophic decline of populations of the most vulnerable species, the damage to other components of communities would vary. Therefore, reliable prediction of the ecological consequences of intense and rare disturbances will require knowledge of the species traits determining disturbance susceptibility, the population recovery processes such as reproduction of surviving individuals and recolonization, and the community importance^26^ of species that are highly susceptible to rare disturbances.

**Figure 2:**
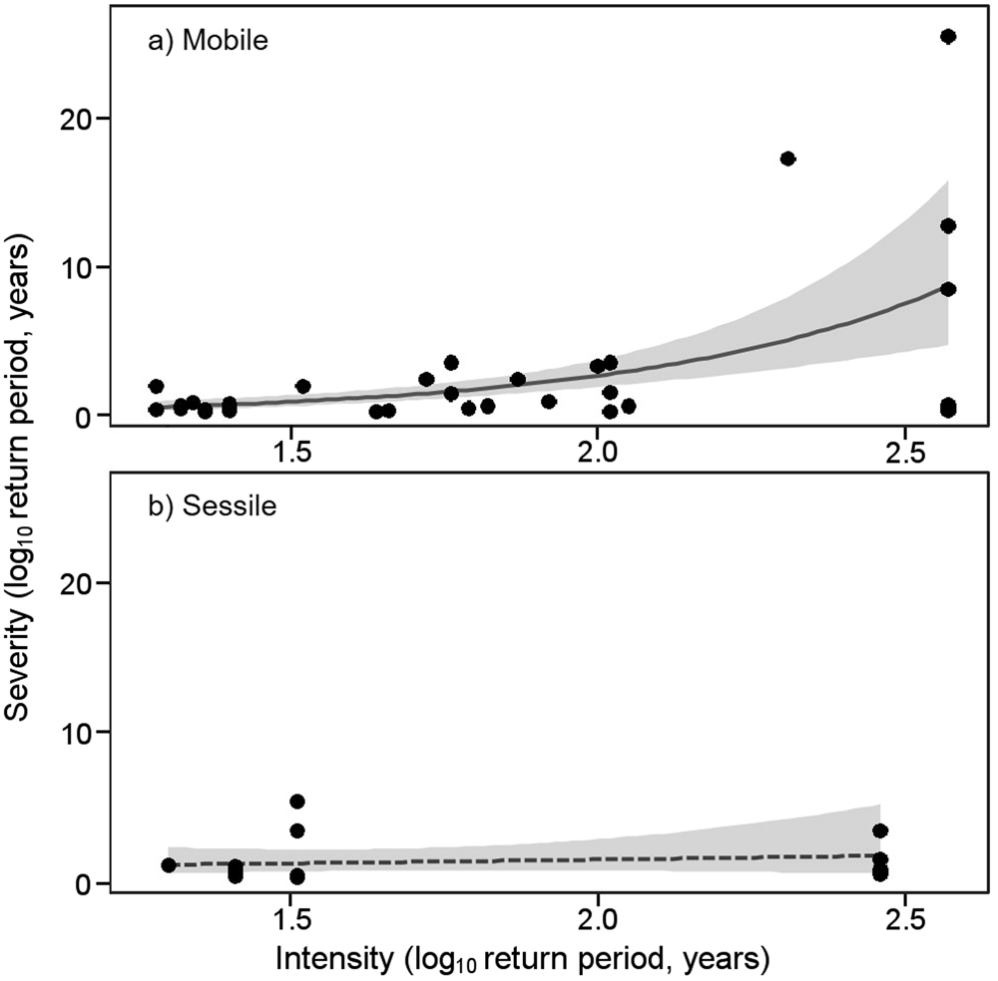
An example of the relationship between intensity and severity in (**a**) mobile and (**b**) sessile groups. Each point is a single species-level severity value. Lines represent the fitted curve estimated by a generalized linear model, with the solid line statistically significant and the dashed line not. Shading demarcates the slope of the 95% confidence intervals.

To demonstrate how the severity–intensity relationship differs among species traits, we used species mobility to split our dataset into two groups and then reanalyzed the data. A significant positive relationship between intensity and severity was obtained for mobile species, but not for sessile ones (Fig. 2). Unfortunately, because of bias in habitat between the two species groups, we could not say that this difference in the severity–intensity relationship depended on the mobility of organisms: all sessile species are rocky intertidal organisms, whereas all mobile species are terrestrial organisms. Therefore, because of the small sample size for each event and bias in the habitats of the species groups, we could not evaluate how the severity–intensity relationship was influenced by event types and species traits in this analysis. In particular, event types are closely related to the habitat and traits (e.g., morphology or life history) of organisms, so that the combination of disturbance types and species traits is likely to have limited variation in the available data. For example, heavy snowfall will likely affect only those species in snowy regions and tsunamis will likely affect only coastal species. Analyzing the effects of event type and species traits on the severity–intensity relationship requires not only an adequate number of samples but also variation in species traits and event types and intensities in each habitat analyzed.

Our findings help to explain a surprising feature of the severity–intensity relationship for a particular event. Unexpectedly, the severity of the mega-tsunami caused by the March 2011 earthquake off the Pacific coast of the Tohoku region was relatively small for rocky intertidal benthic organisms, despite the high intensity of the disturbance^27^ (Fig. 1b). The relatively low severity of the effect of the tsunami might be related to the tsunami’s short duration^28,29^, because the magnitude of the impact of wave force on rocky intertidal benthic organisms is related to the duration^30^. For example, a storm along the Pacific coast of Tohoku; its return period for intensity was 32 years, much shorter than the 290-year return period for the 2011 tsunami (Supplementary Table S1), but the storm’s duration was a few days, much longer than that of the tsunami, a few hours. The severity of the effect of the storm on the same species in the same region of rocky intertidal shore was of the same order as that of the tsunami (Table 1). Thus duration is also an important determinant of the severity of natural disturbance events^31^. Before assessing the effect of duration on severity, we should examine the relationship between intensity and duration. This is because intensity in some disturbances is defined independently of duration, but in others in terms of it. In the former, we can include duration directly in the analysis as an explanatory variable for severity (e.g., earthquakes: seismic intensity). The latter has two cases: when duration is synonymous with intensity (e.g., severe winters: number of days >5-cm snow depth), we cannot include duration in the analysis, but when duration is correlated with intensity (e.g., severe winters: maximum snow depth), we can include a composite variable of duration and intensity obtained by principal component analysis.

**Table 1:** Comparison of the intensity and severity of the impact of a storm in 2006 and a tsunami in 2011 on rocky intertidal benthic organisms in the Tohoku region, Japan.

Event	Intensity (log_10_ return period, years)	Mean severity (±SE) (log_10_ return period, years)
Storm in 2006	1.51	4.79 (±4.78)
Tsunami in 2011	2.46	2.88 (±2.89)

Mean severity is the average of four species-level severity values for each event.

We found that the mean and variance of severity increase exponentially with increasing intensity. From a conservation perspective, it is crucial to predict the severities of physically intense events that occur less than twice in a century and may cause population decline disproportionate to the intensity because of a species’ poor disturbance resistance. However, the severity of the effect of such events cannot be extrapolated from the severity–intensity relationship derived from data for physically weak and frequent disturbances, the population-level consequences of which have been reported extensively^31,32^.

The framework we propose has broad applicability. First, it can be applied at various spatial or temporal scales and for species with various generation times. For example, the disturbance severity can be estimated for more frequent disturbances (e.g., heavy rain) or species with shorter generation times (e.g., microorganisms) at smaller spatial scales by using time series of abundance and physical measures of disturbance recorded at a shorter interval. Second, it can be used to assess the impacts of disturbances on properties at the community level (e.g., species diversity) and ecosystem level (e.g., productivity), as well as at the population level (e.g., population growth rate in this study). In these cases, we can estimate the impact of a disturbance on the focal response property by using time series data on the response property and estimating the return period of the magnitude of change in the value caused by disturbance. Note, however, that the estimated impact at the community and ecosystem levels includes the indirect influence of disturbance. Third, anthropogenic disturbances are also very important and are usually mixed with natural disturbances affecting ecosystems. Nevertheless, it is difficult to quantify the intensity of some anthropogenic disturbances, such as land-use change, pollution, and fishing and harvesting, because such disturbances act irreversibly (e.g., land use), slowly (e.g., pollution), or chronically (e.g., fishing and harvesting), so that our framework for quantifying the severity–intensity relationship is not directly applicable to these disturbances. However, the framework can be used to assess the interactive effect of anthropogenic disturbances and natural disturbances by comparing the severities of natural disturbances between habitats that are affected and not affected by anthropogenic disturbances. Fourth, the severities of most intentional anthropogenic disturbances, such as logging and use of pesticides, can be relatively readily quantified. By considering their severities in the severity–intensity relationship obtained from natural disturbances, we can calculate the intensities of such anthropogenic disturbances by assuming that the severities are comparable to those caused by natural disturbances; such calculations may contribute to the risk management of intentional anthropogenic disturbances.

Deciphering the influence of event types and species traits on the severity–intensity relationship would greatly improve our understanding of the role of natural selection and phylogenetic constraints on evolutionary responses to disturbance. It would also allow us to predict the ecological consequences of various kinds of disturbance events of unexperienced intensity, including extreme weather events caused by climate change^16,33–35^ at multiple ecological levels (e.g., population, community, and ecosystem). Unfortunately, the current lack of studies and the biased datasets make these analyses impossible, highlighting the urgent need to conduct long-term censuses of various kinds of organisms in different habitats.

## Methods

Here, we describe the steps in the analysis of the relationship between the intensity of various types of disturbance and the severity of impact on various species. First, to estimate the severity of impact of various disturbance types on species, we collected high-quality time series of population abundance. Second, by using time series of measured disturbance intensity or historical evidence of a focal disturbance event, we estimated the return period of disturbance intensity in the period during which abundance was surveyed. We then extracted the time series of abundance for populations that experienced a disturbance event with an intensity return period of ≥20 years. Third, we estimated the severity of the impact of the disturbance event by comparing the population growth rate in the year the disturbance occurred with that under normal conditions. Finally, we statistically analyzed the severity–intensity relationship from 27 disturbance events of five types.

### Time series of abundance

To quantify the severity for various species of various disturbance events with a ≥20-year return period of intensity, we obtained primary time series for the abundance of populations affected by such events during the study period (8–42 years) from three sources: (1) Google Scholar; (2) the Global Population Dynamics Database (ver. 2); and (3) our long-term research study of a rocky intertidal shore. The sources and search strategy are described in Supplementary File S1, section 1.

The time series obtained for abundance were pre-processed as needed. From among the pre-processed time series, we then extracted those that satisfied the basic requirements for use in the analysis. Details of the procedures are given in Supplementary File S1, section 1.

### Estimation of disturbance intensity

#### Calculation of return period of intensity

The return period of intensity was calculated by using two different methods, depending on the type of disturbance event, namely (1) a climatic disturbance event (drought, low temperature, severe winter, and storm); or (2) an occasional or rare disturbance event (tsunami) (see Supplementary Figure S1a). For a climatic disturbance event, the physical measure of intensity was recorded annually as the environmental fluctuation, such as the deviation from mean temperature for a severe winter or deviation from mean precipitation for a drought. We statistically estimated the return period of intensity as the inverse of the occurrence probability of the focal disturbance event per year by using long-term time series of force-strength measurements. The occurrence probability of the force strength was assumed to follow either a normal distribution^36^ or a general extreme distribution^15,16^. The occurrence probability of the force strength will follow a normal distribution when the measure is represented by the average or cumulative value (e.g., average temperature or cumulative precipitation). Before assuming a normal distribution, we tested the normality of the data distribution by using the Shapiro–Wilk *W-*test. We log-transformed the data to follow a normal distribution when the assumption was not valid. The occurrence probability of force strength will follow a general extreme distribution when the measure is represented by the maximum or minimum value or a value based on a threshold (e.g., the maximum wind speed or the number of days with >5 cm of snow depth). In both cases, the distribution parameters were estimated from the observed data by using maximum-likelihood estimation. The return period of the event was then calculated from the fitted distribution^36^.

For an occasional or rare disturbance event, the physical measure of the intensity was not recorded annually (e.g., wave height for a tsunami). Therefore, we estimated the return period of intensity as the average interval of occurrence in the reference data of such an event with equal or greater intensity^27^. The interval was estimated by extrapolation based on historical evidence of the event rather than by using direct observational data such as sediment deposits^37^. For each selected time series of abundance, the intensity of the disturbance event was estimated by using the procedure described in Supplementary File S1, section 2.

### Estimation of disturbance severity

#### Calculation of return period of severity and annual population growth rates

For each species, the return period of severity was calculated on the basis of the effect size on the annual population growth rate in the year when the disturbance occurred by using equation (1) for each selected time series of abundance. (See also Supplementary Fig. S1b.) The return period representing a given severity was calculated as the inverse of the occurrence probability of a given effect size, which was statistically estimated assuming a normal distribution.

To estimate the effect size of severity, we calculated the annual population growth rates under normal conditions and those in the year the disturbance event occurred in each selected time series (Supplementary Fig. S1b). To calculate these population growth rates, we obtained 1-year interval time series of abundance, as follows. If multiple abundance measures were made within each year, we selected a single value measured in the same season as the first census conducted after the focal disturbance event. Then abundances with an average value of zero were treated as missing data.

The annual population growth rates under normal conditions, which were defined as those not influenced by the focal disturbance event or other events or stresses such as anthropogenic disturbances, were calculated as follows. First, if it was reported in the original literature that the time series were influenced by such other events or stresses during part of the recording period, we excluded the abundance data during that period from the analysis. Second, if the focal disturbance event influenced the abundance data measured not only immediately after the event but also in subsequent years, we excluded the abundance data during that period from the analysis to minimize, as much as possible, the bias of the indirect influence of the event on estimated severity. For example, an exponential increase in abundance subsequent to a population decline can be indirectly caused by the disturbance event. To detect the temporal trend in abundance that seemed to be caused by the indirect effect of the focal disturbance event in part of the second and subsequent annual abundance data collected after the event, we examined the relationship between abundance and year by using linear regression. If the temporal trend in abundance was significant (*P* < 0.05), we excluded data influenced by the indirect effect of the focal disturbance event by removing data beginning with the oldest census; we included in the analysis only the part of the annual abundance data that did not represent the significant temporal trend.

The population growth rate in the year the disturbance event occurred was calculated by using the two abundance measures recorded immediately before and after the disturbance event. If a disturbance event drives the mean abundance of a population to zero immediately after the event, then the effect size, as the basis of severity, should be calculated by using the abundance plus a small number in order to avoid an infinite value (i.e., to prevent division by zero); however, the database we used for the meta-analysis did not include such cases. The detailed procedures to estimate the severity of the effect of a disturbance are described in Supplementary File S1, section 3.

### Statistical analysis

The relationship between log_10_ of the return periods of intensity and severity was analyzed by using a generalized linear model, taking severity as the response variable and intensity as the explanatory variable. The error distribution and link function in the generalized linear model were based on the value of Akaike’s information criterion and the Schwarz Bayesian information criterion for each potential model (the model with the smallest value was chosen); this resulted in gamma-distributed errors with a log-link function (see Supplementary Table S4). All analyses were performed with version 3.02 of the R programming language (www.r-project.org)^38^.

## Electronic supplementary material

Supplementary Information
