# Supplementary material for: A framework for quantifying the relationship between intensity and severity of impact of disturbance across types of events and species
Source: Sci Rep. 2018 Jan 15;8:795. doi: 10.1038/s41598-017-19048-5 (PMC5768861; doi:10.1038/s41598-017-19048-5)
Supplement: Supplementary file 1 — Supplementary Information [file 41598_2017_19048_MOESM1_ESM.pdf]

Online supplement

# A framework for quantifying the relationship between intensity and severity of impact of disturbance across types of events and species

Aiko Iwasaki[1]* and Takashi Noda[2]

[1] Graduate School of Environmental Science, Hokkaido University, N10W5, Kita-ku, Sapporo, Hokkaido 060-0810, Japan

[2] Faculty of Environmental Science, Hokkaido University, N10W5, Kita-ku, Sapporo, Hokkaido 060-0810, Japan

* Corresponding author. E-mail: aiwasaki@ees.hokudai.ac.jp, Tel.: 81-11-706-2245

**Supplementary File S1| Detailed description of methods**

## 1. Time series of abundance

*Sources of time series of abundance*

To quantify the severity of the impact of various disturbance events with an intensity return period of ≥20 years on various species, we obtained primary time series for the abundance of populations affected by such an event during the study period from three sources: (1) Google Scholar (GS); (2) the Global Population Dynamics Database (GPDD, ver. 2); and (3) our research project along a rocky intertidal shore (RPRI). When searching GS, to gather primary literature by authors self-identifying and reporting the effects of disturbance events on populations we used a keyword search for each of the following terms or their combinations: ("abundance" OR "population size") AND ("natural disturbance" OR "catastrophic event" OR "extreme climate") AND ("impact" OR "decrease" OR "damage"), ("long term"), ("large scale" OR "region*"), ("pre and post" OR "before and after"). We finished conducting our search on 24 December 2014; the search yielded 2940 hits. GPDD is the largest database of population time series and includes >10-year time series for nearly 5000 populations of 1800 species (24 classes, including terrestrial plants, e.g., Dicotyledoneae; terrestrial and aquatic invertebrates, e.g., Insecta and Bivalvia; terrestrial and aquatic vertebrates, e.g., Mammalia and Osteichthyes; and unicellular organisms, e.g., Dinophyceae and Bacillariophyceae) across the globe. In the case of the abundance data obtained from GS and GPDD, we examined whether the time series met requirements for analysis by reading the original papers or books in the subsequent selection processes; we excluded time series for which the literature was not available. RPRI provided 12 years of time series for hundreds of populations of sessile organisms on rocky intertidal shores (e.g., algae, barnacles, and bivalves) in six regions along the Pacific coast of Japan. Within each region (>10 $km^2$) we chose five shores along the coastline. Within each shore, we established five census plots. Each plot was 50 cm wide by 100 cm high, and the mean tidal level corresponded to the vertical midpoint of the plot on steep rock walls. Detailed descriptions of the study sites and biogeographic features of the area can be found in previous reports[1,2].

*Pre-processing of the resulting time series of abundance*

The resulting time series of abundance were pre-processed, as needed, to provide a single time series of population abundance that included data measured regularly at 1-year or shorter intervals for each species in each study. If the time series of abundance was represented as plots or graphs, we measured values from the figure by applying

quantitative methods to highly magnified images using GSYS2.4 (www.jcprg.org/gsys/2.4/index-j.html). If there were multiple census points within the study site, we used the average abundance across the site. If the recording periods differed among the census points within the study site, we used the data in the period for which (1) the census was conducted at multiple census points and (2) the combination of the census points was consistent.

*Quality assessment of the pre-processed time series of abundance and selection of the target species*

To extract the time series of abundance that satisfied the basic requirement for use in the analysis, we selected relevant time series from the pre-processed ones on the basis of (1) quality assessment of the time series of abundance and (2) selection of the target species. Quality of the time series was assessed on the basis of the purpose of the study, the resolution of the census, the measurement of abundance, the temporal trend of abundance, the spatial scale of the time series, and the length of the time series (Supplementary Table S3-i online). Target species were selected on the basis of the absence of seasonal migration and on generation time (Supplementary Table S3-ii online). The generation time condition for analysis was determined by the temporal scale of the disturbance. We identified disturbance events with a ≥20-year return period with regard to intensity, so we used species with generation times of ≤10 years, because an event that occurs less than once in two generations can be assumed to be a deviation from the usual environmental fluctuation. Although it would be ideal to equalize the relative length of the return period to the generation time of each species, we could not do this owing to the lack of information on the exact generation times of some species and the limited sample numbers.

## 2. Estimation of disturbance intensity

For each selected time series of abundance, the intensity of a disturbance event that appeared to decrease the focal species abundance and occurred in the period of the study was estimated by using different procedures, depending on whether the disturbance event had been reported in the original literature of the focal time series (I) or not (II) (see Supplementary Fig. S2-I and Fig. S2-II).

*I. Estimation of disturbance event intensity when the event was reported in the original literature (Fig. S2-I).*

When the time series of force-strength measurement was reported in the original literature (Fig. S2-I, A), we estimated the distribution of the occurrence probability of

the force-strength measurement and then identified the physical intensity of the focal disturbance event reported in the original literature. Next, we estimated the return period of the disturbance intensity as the inverse of the occurrence probability of the focal disturbance event per year. Finally, if the return period of the intensity of the focal disturbance event was ≥20 years, we selected the disturbance event for analysis.

When the time series of force-strength measurement was not reported in the original literature (Fig. S2-I, B), we used the following two procedures, depending on whether the focal disturbance was a climatic disturbance event (Fig. S2-I, C) or an occasional or rare disturbance event (Fig. S2-I, D). For a climatic disturbance event, we obtained the time series of the climatic parameter as the force-strength measurements recorded at the station nearest (<20 km distance) to the focal study site. In climatic disturbance events, the climatic parameter (e.g., snowfall for severe winters and wave height for storms) likely has values similar to those at the study site and the nearby station[3,4]. We selected and used the time series of the climatic parameter that had been recorded at stations with topographic and geographic conditions similar to those of the site (e.g., flatland or distance from the sea) and that covered the whole period for which the population abundance survey was conducted. If there were multiple available time-series data, we used their average value. After these procedures, we estimated the distribution of occurrence probabilities of the climatic parameter. We then identified the intensity of the disturbance event that occurred in the period when the population abundance survey was conducted and estimated the return period of the disturbance intensity. For an occasional or rare disturbance event, we obtained historical evidence of the focal disturbance event from other literature. We then estimated the return period of intensity of the disturbance event as the average interval of occurrence of such an event with equal or greater intensity. In both procedures, we selected for analysis those disturbance events with an intensity return period of ≥20 years.

*II. Estimation of disturbance event intensity when no disturbance event was reported in the original literature (Fig. S2-II).*

When a disturbance event was not reported in the original literature (Fig. S2-II, A), we identified whether the time series of environmental parameters represented the force-strength of the disturbance. In such cases, we estimated the distribution of occurrence probabilities of the force-strength measurements. We then estimated the force-strength of the disturbance for each year in the period when the population abundance survey was conducted. When the environmental parameters were ones that did not represent force-strength, we identified the main type of disturbance event for the focal species by reading the original report or other literature. When the focal disturbance event was

climatic (Fig. S2-II, C), before we estimated the distribution of occurrence probabilities of the force-strength measurement, we obtained the time series of climatic parameters measured at the station nearest to the study site. When the focal disturbance event was rare or occasional (Fig. S2-II, D), before we estimated the return period of the disturbance intensity, we obtained historical evidence of the focal disturbance event from other literature. In both procedures, we selected for analysis those disturbance events with intensity return periods of ≥20 years.

If the time series of the environmental parameters had not been reported in the original literature (Fig. S2-II, B), we identified the main type of disturbance event for the focal species by reading the original report or other literature. The following steps were the same as in Fig. S2-II, A and the subsequent procedure.

**3. Estimation of disturbance severity**

To estimate the severity of the impact of a disturbance, we first selected the time series of abundance that satisfied the minimum requirements for calculation of the severity (Table S3-iii). Next, we calculated the effect size representing the severity. Finally, we excluded those data for which the estimated effect size was positive, meaning that the annual population growth rate in the year of the disturbance event was higher than usual. The direct effect of a disturbance event cannot be an increase in population abundance, so a positive effect may indicate that the indirect effect of the event was too large to allow evaluation of the direct effect of the focal disturbance event.

**References**


1. Nakaoka, M., Ito, N., Yamamoto, T., Okuda, T. & Noda, T. Similarity of rocky intertidal assemblages along the Pacific coast of Japan: effects of spatial scales and geographic distance. *Ecol. Res.* **21**(3), 425–435 (2006).
2. Okuda, T., Noda, T., Yamamoto, T., Ito, N. & Nakaoka, M. Latitudinal gradient of species diversity: multi-scale variability in rocky intertidal sessile assemblages along the Northwestern Pacific coast. *Popul. Ecol.* **46**(2), 159–170 (2004).
3. Perry, M. & Hollis, D. The generation of monthly gridded datasets for a range of climatic variables over the UK. *Int. J. Climatol.* **25**(8), 1041–1054 (2005).
4. Izaguirre, C., Méndez, F. J., Menéndez, M. & Losada, I. J. Global extreme wave height variability based on satellite data. *Geophys. Res. Lett.* **38**(10), 1–6 (2011).

## a) Estimation of intensity

| Type of event | Type of data used for calculation | Formula of return period of intensity | Distribution type followed by occurrence probability | Example of data |
|---|---|---|---|---|
| Climatic event (e.g., severe winter) | Time series of climatic parameter | $\frac{1}{\text{Occurrence probability}}$ | Normal distribution 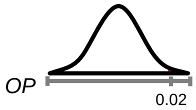 | Total snowfall |
| | | | General extreme distribution 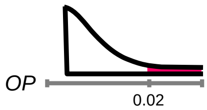 | Maximum snow depth |
| Rare or occasional event (e.g., tsunami) | Historical evidence | $\frac{\text{Length of recording period}}{\text{number of such events}}$ | – | Tsunami height |

## b) Estimation of severity

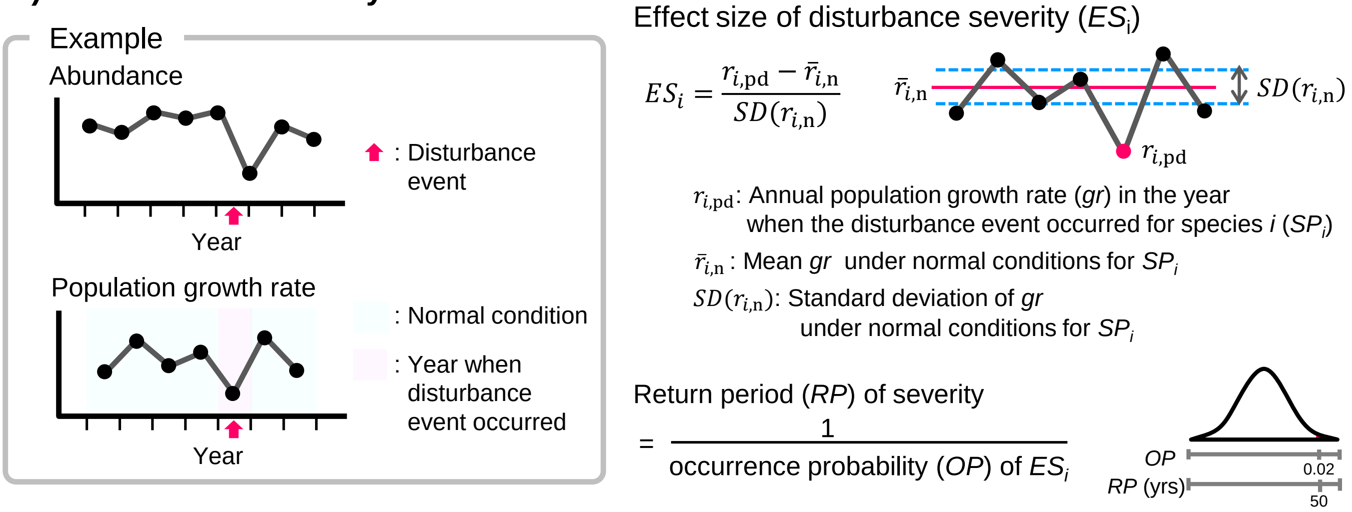


**Supplementary Figure S1| Diagram of estimation of intensity and severity.** (a) Intensity was estimated by two different methods depending on the type of disturbance event: climatic events and rare or occasional events. Intensity was represented by the return period, which was estimated as the inverse of the occurrence probability for climatic events and as the mean interval time of the focal disturbance event with equal or greater magnitude for rare or occasional events. (b) Severity estimation was based on the effect size, which was calculated by comparing the population growth rate between the year when the disturbance event occurred and under normal conditions. Severity was represented by the return period, which was estimated as the inverse of the occurrence probability of the effect size.

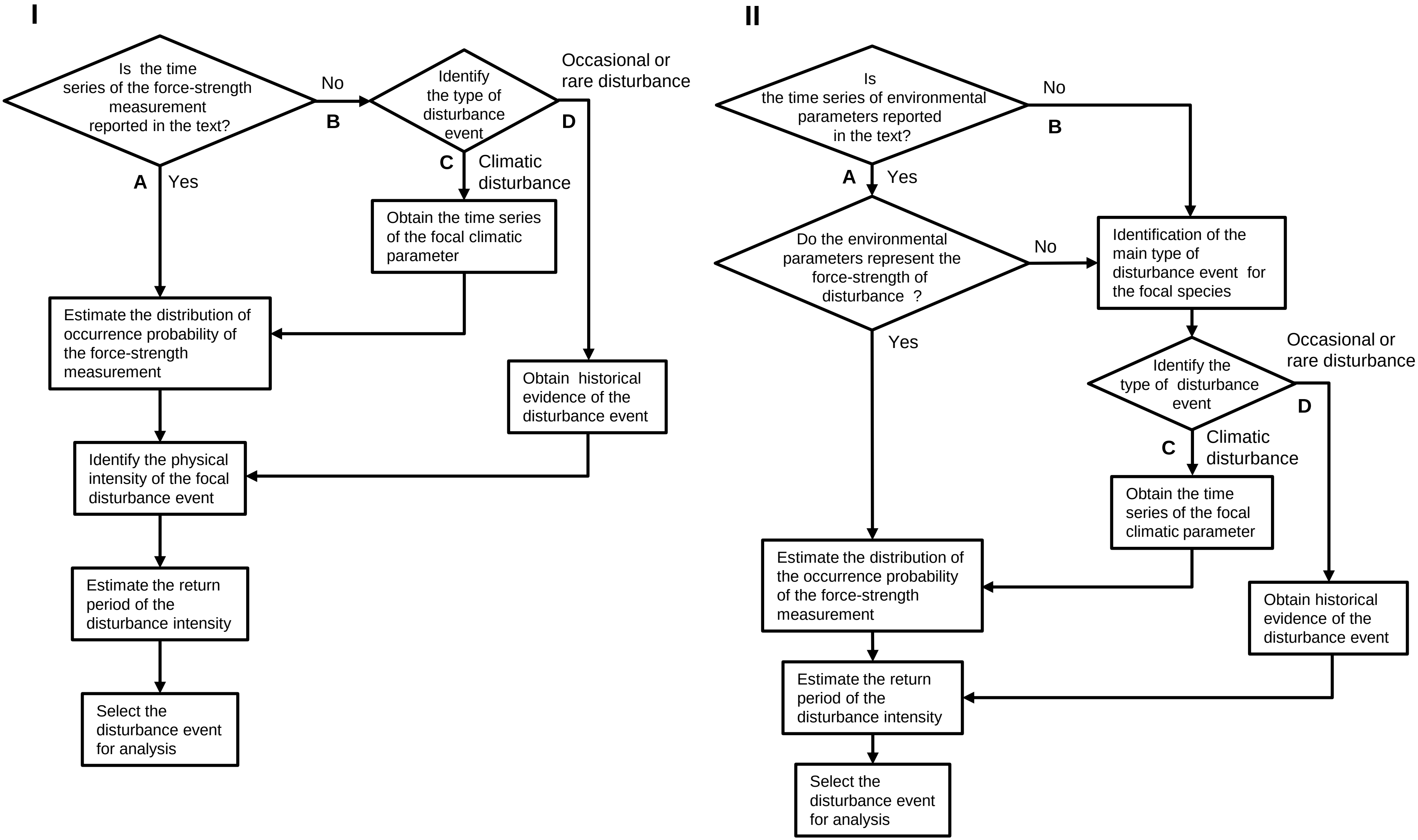


**Supplementary Figure S2| Flow chart of the estimation of intensity of a disturbance event.**
Two procedures used to estimate the intensity of the disturbance event that seemed to decrease the focal species abundance and occurred in the period of the study, for each time series: (I) when the disturbance event was reported in the original literature and (II) when no disturbance event was reported.

**Table S1| Data used for the meta-analysis**

Information about the disturbance events and species affected by those events. NOAA: U.S. National Oceanic and Atmospheric Administration; JMA: Japan Meteorological Association; GPDD: Global Population Dynamics Database (ver. 2); GS: Google Scholar; RSRI: authors' research study on a in rocky intertidal shore.

| | | Disturbance Event | | | | | | | | Severity | | | | | | | | | | |
|---|---|---|---|---|---|---|---|---|---|---|---|---|---|---|---|---|---|---|---|---|
| No. | Event No. | Year | Type of event | Force-strength measurement | Distribution of force-strength measurement | Length of record (years) | Return period of intensity ($log_{10}$ years) | Data source | Record region | Species | Taxon (Class) | Habitat | Species trait | Normal period (years) | $ES_i$ | 95% CI of $ES_i$ (lower, upper) | Return period ($log_{10}$ years) | Time series source | Reference | Notes |
| 1 | 1 | 1946 | Severe winter | Winter minimum temperature | Extreme | 107 | 1.76 | Met Office | Central England | *Ardea cinerea* | Aves | Forest | Mobile | 11 | 3.47 | (2.13, 5.04) | 3.58 | GPDD | Lack (1954) | |
| 2 | 2 | 1947 | Severe winter | Maximum snow depth | Extreme | 72 | 1.34 | NOAA | Malcolm, Nebraska, US<br>Bennet, Nebraska, US<br>Raymond 2 E, Nebraska, US<br>Lincoln University Power Plant, Nebraska, US | *Colinus virginianus* | Aves | Water system | Mobile | 40 | 1.15 | (0.84, 1.45) | 0.90 | GPDD | Nebraska Game and Parks Commission (1990) | |
| 3 | 3 | 1983 | Severe winter | Maximum snow depth | Extreme | 72 | 1.92 | NOAA | Malcolm, Nebraska, US<br>Bennet, Nebraska, US<br>Raymond 2 E, Nebraska, US<br>Lincoln University Power Plant, Nebraska, US | *Colinus virginianus* | Aves | Water system | Mobile | 40 | 1.26 | (0.94, 1.57) | 0.98 | GPDD | Nebraska Game and Parks Commission (1990) | |
| 4 | 4 | 1959 | Severe winter | Maximum snow depth | Extreme | 72 | 1.87 | NOAA | Malcolm, Nebraska, US<br>Bennet, Nebraska, US<br>Raymond 2 E, Nebraska, US<br>Lincoln University Power Plant, Nebraska, US | *Colinus virginianus* | Aves | Water system | Mobile | 40 | 2.74 | (2.26, 3.28) | 2.51 | GPDD | Nebraska Game and Parks Commission (1990) | |
| 5 | 5 | 1951 | Severe winter | Number of snow (>5 cm) days | Extreme | 57 | 1.36 | The original paper | | *Tyto alba* | Aves | Forest | Mobile | 31 | 0.35 | (-0.11, 0.88) | 0.44 | GS | Altwegg et al. (2006) | |
| 6 | 6 | 1952 | Severe winter | Number of snow (>5 cm) days | Extreme | 57 | 1.72 | The original paper | | *Tyto alba* | Aves | Forest | Mobile | 31 | 2.71 | (1.46, 4.28) | 2.47 | GS | Altwegg et al. (2006) | |
| 7 | 7 | 1986 | Severe winter | Number of snow (>5 cm) days | Extreme | 57 | 1.32 | The original paper | | *Tyto alba* | Aves | Forest | Mobile | 31 | 0.63 | (0.08, 1.28) | 0.58 | GS | Altwegg et al. (2006) | |
| 8 | 8 | 1976 | Severe winter | Number of snow (>5 cm) days | Extreme | 57 | 1.32 | The original paper | | *Tyto alba* | Aves | Forest | Mobile | 31 | 0.80 | (0.19, 1.50) | 0.67 | GS | Altwegg et al. (2006) | |
| 9 | 9 | 1962 | Severe winter | Number of snow (>5 cm) days | Extreme | 57 | 2.00 | The original paper | | *Tyto alba* | Aves | Forest | Mobile | 31 | 3.33 | (1.86, 5.22) | 3.36 | GS | Altwegg et al. (2006) | |
| 10 | 10 | 1956 | Low temperature | Annual mean temperature | Normal | 137 | 2.02 | NOAA | Bamberg Germany | *Bupalus piniaria* | Insecta | Forest | Mobile | 13 | 1.99 | (1.42, 2.80) | 1.63 | GPDD | Klimetzek (1988) | Bavaria |
| 11 | 10 | 1956 | Low temperature | Annual mean temperature | Normal | 137 | 2.02 | NOAA | Bamberg, Germany | *Diprion pini* | Insecta | Forest | Mobile | 16 | 0.04 | (-0.58, 0.56) | 0.32 | GPDD | Klimetzek (1988) | Bavaria |
| 12 | 10 | 1956 | Low temperature | Annual mean temperature | Normal | 137 | 2.02 | NOAA | Bamberg, Germany | *Panolis flammea* | Insecta | Forest | Mobile | 16 | 3.49 | (2.08, 5.52) | 3.62 | GPDD | Klimetzek (1988) | Bavaria |
| 13 | 11 | 1986 | Low temperature | Average water temperature | Normal | 106 | 1.30 | The original paper | | *Semibalanus balanoides* | Crustacea | Intertidal shore | Sessile | 31 | 1.45 | (0.94, 2.08) | 1.13 | GS | Hawkins et al. (2003) | |
| 14 | 12 | 1962 | Low temperature | Average temperature in Mar-May | Normal | 106 | 1.66 | Met Office | United Kingdom | *Parus major* | Aves | Forest | Mobile | 15 | 0.21 | (-0.35, 0.68) | 0.38 | GPDD | O'Connor (1980) | |
| 15 | 13 | 1962 | Severe winter | Mean temperature of winter (Dec-Mar) | Normal | 105 | 2.57 | NOAA | United Kingdom | *Alectoris rufa* | Aves | Farm land | Mobile | 12 | 0.23 | (-0.47, 0.72) | 0.39 | GPDD | Batten & Marchant (1977) | |
| 16 | 13 | 1962 | Severe winter | Mean temperature of winter (Dec-Mar) | Normal | 105 | 2.57 | NOAA | United Kingdom | *Perdix perdix* | Aves | Farm land | Mobile | 12 | 0.93 | (0.35, 1.51) | 0.75 | GPDD | Batten & Marchant (1977) | |
| 17 | 13 | 1962 | Severe winter | Mean temperature of winter (Dec-Mar) | Normal | 105 | 2.57 | NOAA | United Kingdom | *Vanellus vanellus* | Aves | Farm land | Mobile | 8 | 5.82 | (3.53, 11.13) | 8.53 | GPDD | Batten & Marchant (1977) | |
| 18 | 13 | 1962 | Severe winter | Mean temperature of winter (Dec-Mar) | Normal | 105 | 2.57 | NOAA | United Kingdom | *Gallinula chloropus* | Aves | Farm land | Mobile | 11 | 10.53 | (7.32, 15.35) | 25.50 | GPDD | Batten & Marchant (1977) | |
| 19 | 13 | 1962 | Severe winter | Mean temperature of winter (Dec-Mar) | Normal | 105 | 2.57 | NOAA | United Kingdom | *Carduelis carduelis* | Aves | Farm land | Mobile | 11 | 0.75 | (0.08, 1.52) | 0.64 | GPDD | Batten & Marchant (1977) | |
| 20 | 13 | 1962 | Severe winter | Mean temperature of winter (Dec-Mar) | Normal | 105 | 2.57 | NOAA | United Kingdom | *Motacilla alba* | Aves | Farm land | Mobile | 10 | 7.29 | (4.62, 11.27) | 12.81 | GPDD | Batten & Marchant (1977) | |
| 21 | 14 | 1965 | Severe winter | Mean temperature of winter (Dec-Mar) | Normal | 50 | 1.82 | NOAA | Alajarvi Moksy 1, Finland | *Sorex araneus* | Mammalia | Forest | Mobile | 9 | 0.81 | (-0.02, 1.64) | 0.68 | GPDD | Henttonen et al. (1989) | Eastern Finland |
| 22 | 15 | 1968 | Severe winter | Annual maximum snow accumulation | Extreme | 28 | 1.76 | The original paper | | *Alces alces* | Mammalia | Forest | Mobile | 24 | 1.89 | (1.39, 2.52) | 1.53 | GS | Messier (1991) | |
| 23 | 16 | 1969 | Severe winter | Total snow precipitation | Normal | 18 | 1.28 | The original paper | | *Aythya americana* | Aves | Wetland | Mobile | 16 | 0.43 | (-0.22, 0.94) | 0.48 | GPDD | Higgins et al. (1992) | |
| 24 | 16 | 1969 | Severe winter | Total snow precipitation | Normal | 18 | 1.28 | The original paper | | *Oxyuya jamaicensis* | Aves | Wetland | Mobile | 16 | 2.35 | (1.64, 3.24) | 2.03 | GPDD | Higgins et al. (1992) | |
| 25 | 17 | 1979 | Severe winter | Total snow precipitation | Normal | 18 | 1.40 | The original paper | | *Anas discors* | Aves | Wetland | Mobile | 16 | 1.10 | (0.38, 1.85) | 0.87 | GPDD | Higgins et al. (1992) | |
| 26 | 17 | 1979 | Severe winter | Total snow precipitation | Normal | 18 | 1.40 | The original paper | | *Anas clypeata* | Aves | Wetland | Mobile | 16 | 0.77 | (0.26, 1.38) | 0.66 | GPDD | Higgins et al. (1992) | |
| 27 | 17 | 1979 | Severe winter | Total snow precipitation | Normal | 18 | 1.40 | The original paper | | *Aythya americana* | Aves | Wetland | Mobile | 16 | 0.33 | (-0.30, 0.83) | 0.43 | GPDD | Higgins et al. (1992) | |
| 28 | 17 | 1979 | Severe winter | Total snow precipitation | Normal | 18 | 1.40 | The original paper | | *Oxyuya jamaicensis* | Aves | Wetland | Mobile | 16 | 0.65 | (0.09, 1.20) | 0.59 | GPDD | Higgins et al. (1992) | |
| 29 | 18 | 1971 | Severe winter | Mean temperature of winter (Dec-Mar) | Normal | 70 | 1.79 | NOAA | Haines Junction, California, US | *Ovis dalli dalli* | Mammalia | Boreal forest and shrub | Mobile | 10 | 0.57 | (-0.07, 1.30) | 0.55 | GPDD | Hoefs & Bayer (1983) | |
| 30 | 19 | 1984 | Low temperature | Average temperature in spring | Normal | 68 | 2.05 | The original paper | | *Lygacus equestris* | Insecta | Grassland | Mobile | 7 | 0.81 | (0.02, 2.35) | 0.68 | GS | Solbreck (1991) | |
| 31 | 20 | 1987 | Low temperature | Average temperature in spring | Normal | 68 | 2.31 | The original paper | | *Lygacus equestris* | Insecta | Grassland | Mobile | 7 | 8.56 | (6.63, 13.58) | 17.25 | GS | Solbreck (1991) | |
| 32 | 21 | 1989 | Drought | Annual precipitation | Normal | 119 | 1.52 | The original paper | | *Euura lasiolepis* | Insecta | Forest | Mobile | 13 | 2.35 | (1.71, 3.25) | 2.03 | GS | Hawkins & Holyoak (1998) | Arizona |
| 33 | 22 | 1985 | Drought | Annual precipitation | Normal | 119 | 1.36 | The original paper | | *Coleophora laricella* | Insecta | Forest | Mobile | 8 | 0.13 | (-0.71, 0.81) | 0.35 | GS | Hawkins & Holyoak (1998) | Oregon |
| 34 | 23 | 1985 | Drought | Annual precipitation | Normal | 19 | 1.64 | The original paper | | *Malacosoma pluviale* | Insecta | Forest | Mobile | 10 | 0.03 | (-0.69, 0.82) | 0.31 | GS | Hawkins & Holyoak (1998) | Victoria, BC |
| 35 | 24 | 2006 | Storm | Annual maximum wave height | Extreme | 18 | 1.41 | JMA | Tokachi, Japan | *Pterosiphonia bipinnata* | Phaeophyceae | Intertidal shore | Sessile | 8 | 0.40 | (-0.41, 1.10) | 0.46 | RSRI | Noda (unpublished data) | Doto |
| 36 | 24 | 2006 | Storm | Annual maximum wave height | Extreme | 18 | 1.41 | JMA | Tokachi, Japan | *Chthamalus challengeri* | Crustacea | Intertidal shore | Sessile | 11 | 0.22 | (-0.50, 0.83) | 0.38 | RSRI | Noda (unpublished data) | Doto |
| 37 | 24 | 2006 | Storm | Annual maximum wave height | Extreme | 18 | 1.41 | JMA | Tokachi, Japan | *Neosiphonia yendoi* | Chlorophyceae | Intertidal shore | Sessile | 11 | 0.54 | (-0.17, 1.64) | 0.53 | RSRI | Noda (unpublished data) | Doto |
| 38 | 24 | 2006 | Storm | Annual maximum wave height | Extreme | 18 | 1.41 | JMA | Tokachi, Japan | *Gloiopeltis furcata* | Florideophyceae | Intertidal shore | Sessile | 11 | 0.85 | (0.00, 1.78) | 0.70 | RSRI | Noda (unpublished data) | Doto |
| 39 | 25 | 2006 | Storm | Annual maximum wave height | Extreme | 28 | 1.51 | JMA | Kamaishi, Japan | *Chthamalus challengeri* | Crustacea | Intertidal shore | Sessile | 6 | 0.31 | (-0.69, 2.69) | 0.42 | RSRI | Noda (unpublished data) | Sanriku |
| 40 | 25 | 2006 | Storm | Annual maximum wave height | Extreme | 28 | 1.51 | JMA | Kamaishi, Japan | *Corallina pilulifera* | Florideophyceae | Intertidal shore | Sessile | 6 | 0.05 | (-2.50, 1.33) | 0.32 | RSRI | Noda (unpublished data) | Sanriku |
| 41 | 25 | 2006 | Storm | Annual maximum wave height | Extreme | 28 | 1.51 | JMA | Kamaishi, Japan | *Crassostrea gigas* | Bivalvia | Intertidal shore | Sessile | 6 | 4.46 | (3.06, 6.99) | 5.39 | RSRI | Noda (unpublished data) | Sanriku |
| 42 | 25 | 2006 | Storm | Annual maximum wave height | Extreme | 28 | 1.51 | JMA | Kamaishi, Japan | *Septifer virgatus* | Bivalvia | Intertidal shore | Sessile | 5 | 3.40 | (2.48, 6.32) | 3.47 | RSRI | Noda (unpublished data) | Sanriku |
| 43 | 26 | 2006 | Storm | Annual maximum wave height | Extreme | 18 | 1.41 | JMA | Tokachi, Japan | *Ralfsia verrucosa* | Phaeophyceae | Intertidal shore | Sessile | 9 | 1.29 | (0.40, 2.68) | 1.01 | RSRI | Noda (unpublished data) | Donan |
| 44 | 26 | 2006 | Storm | Annual maximum wave height | Extreme | 18 | 1.41 | JMA | Tokachi, Japan | *Leathesia marina* | Phaeophyceae | Intertidal shore | Sessile | 10 | 0.73 | (0.05, 1.57) | 0.63 | RSRI | Noda (unpublished data) | Donan |
| 45 | 26 | 2006 | Storm | Annual maximum wave height | Extreme | 18 | 1.41 | JMA | Tokachi, Japan | *Hildenbrandia rubra* | Florideophyceae | Intertidal shore | Sessile | 10 | 1.01 | (0.12, 1.78) | 0.81 | RSRI | Noda (unpublished data) | Donan |
| 46 | 26 | 2006 | Storm | Annual maximum wave height | Extreme | 18 | 1.41 | JMA | Tokachi, Japan | *Septifer virgatus* | Phaeophyceae | Intertidal shore | Sessile | 10 | 1.09 | (0.39, 1.79) | 0.86 | RSRI | Noda (unpublished data) | Donan |
| 47 | 27 | 2011 | Tsunami | Tsunami deposit | - | 4000 | 2.46 | Ishimura & Miyauchi (2015) [23] | | *Chthamalus challengeri* | Crustacea | Intertidal shore | Sessile | 6 | 0.51 | (-0.50, 2.76) | 0.52 | RSRI | Noda (unpublished data) | |
| 48 | 27 | 2011 | Tsunami | Tsunami deposit | - | 4000 | 2.46 | Ishimura & Miyauchi (2015) [23] | | *Corallina pilulifera* | Florideophyceae | Intertidal shore | Sessile | 6 | 1.00 | (0.41, 1.53) | 0.80 | RSRI | Noda (unpublished data) | |
| 49 | 27 | 2011 | Tsunami | Tsunami deposit | - | 4000 | 2.46 | Ishimura & Miyauchi (2015) [23] | | *Crassostrea gigas* | Bivalvia | Intertidal shore | Sessile | 6 | 1.83 | (1.10, 2.90) | 1.47 | RSRI | Noda (unpublished data) | |
| 50 | 27 | 2011 | Tsunami | Tsunami deposit | - | 4000 | 2.46 | Ishimura & Miyauchi (2015) [23] | | *Septifer virgatus* | Bivalvia | Intertidal shore | Sessile | 5 | 3.40 | (2.48, 6.32) | 3.47 | RSRI | Noda (unpublished data) | |

**Supplementary Table S2| References used in the meta-analysis**

A full listing of the 14 published literature and electronic sources used in the meta-analysis of the severity of disturbance events.


1. Altwegg, R., Roulin, A., Kestenholz, M. & Jenni L. Demographic effects of extreme winter weather in the barn owl. *Oecologia* **149**, 44-51 (2006).
2. Batten, L. A. & Marchant, J. H. Bird population changes for the years 1974–75. *Bird Study* **24**, 55-61 (1977).
3. Hawkins, B. A. & Holyoak, M. Transcontinental crashes of insect populations? *Am. Nat.* **152**, 480-484 (1998).
4. Hawkins, S. J., Southward, A. J. & Genner, M. J. Detection of environmental change in a marine ecosystem: evidence from the western English Channel. *Sci. Total Environ.* **310**, 245-256 (2003).
5. Henttonen, H. *et al.* Long-term population dynamics of the common shrew *Sorex araneus* in Finland. *Ann. Zool. Fenn.* **26**, 349-355 (1989).
6. Higgins, K. F., Kirsch, L. M., Klett, A. T. & Miller, H. W. in *Waterfowl Production on the Woodworth Station in South-central North Dakota, 1965-1981* (U.S. Fish and Wildlife Service Resource Publication 180, 1992).
7. Hoefs, M. & Bayer, M. Demographic characteristics of an unhunted Dall sheep (*Ovis dalli dalli*) population in southwest Yukon, Canada. *Can. J. Zool.* **61**, 1346-1357 (1983).

8. Klimetzek, D. Population dynamics of pine-feeding insects: a historical study. in *Population Dynamics of Forest Insects* (eds. Watt, A. D., Leather, S. R., Hunter, M. D. & Kidd, N. A. C.) 3-10 (Intercept, Andover, UK, 1990).
9. Lack, D. The comparative stability of population in *The Natural Regulation of Animal Numbers*, 7-20 (Claredon Press, Oxford, 1954).
10. Messier, F. The significance of limiting and regulating factors on the demography of moose and white-tailed deer. *J. Anim. Ecol.* **60**, 377-393 (1991).
11. Nebraska Game and Parks Commission. Wild Life Species Guide, Bobwhie Quail. http://outdoor.nebraska.ne.gov/wildlife/wildlife_species_guide/quail.asp (accessed 22 Oct 2015).
12. O'Connor, R. J. Pattern and process in great tit (*Parus major*) populations in Britain. *Ardea* **68**, 165-183 (1980).
13. Solbreck, C. Unusual weather and insect population dynamics: *Lygaeus equestris* during an extinction and recovery period. *Oikos* **60**, 343-350 (1991).
14. Stafford, J. The heron population of England and Wales, 1928–1970. *Bird Study* **18**, 218-221 (1971).

**Supplementary Table S3| Selection criteria of time series of abundance for use in analysis**

| Process | Category | Criteria of time series of abundance | | Reason |
|---|---|---|---|---|
| | | Type | Description | |
| i) Quality of time series of abundance | Purpose of study | Exclusion | Obtained for purposes other than estimation of wild population size | To exclude the effects of anthropogenic influence(s) such as fishing and hunting on abundance |
| | Resolution of abundance | Exclusion | Combined abundances of several species | To evaluate species-level severity |
| | Measure of abundance | Exclusion | Represented by relative abundance and indirect measurement | To ensure accuracy of the population dynamics of focal species |
| | Temporal trend | Exclusion | Representing significant ($P$<0.05) temporal trend in abundance by linear regression | To eliminate data on populations influenced by chronic stress other than the focal disturbance event during the whole recording period |
| | Spatial scale of study | Inclusion | Obtained by census conducted at a scale $\geq$10 $km^2$ | To accurately estimate the general picture of the event's severity at a sufficient spatial scale relative to the scale of the event [1] [2] |
| | Length of time series | Exclusion | Length less than twice the generation time | To ensure accuracy of the population dynamics of the focal species and severity estimation by using sufficient length of time series relative to generation time of focal species [3] |
| ii) Target species | Seasonal migration | Exclusion | Observing migratory species | Difficulty in specifying the place and timing of population decline and the factor(s) causing it |
| | Generation time | Exclusion | Observing species with generation times <1 year | To avoid underestimation of severity caused by growth and reproduction occurring immediately after the disturbance |
| | Generation time | Exclusion | Observing species with generation times >10 years | To target the species that seem to experience the focal disturbance event less than once in two generations |
| iii) Requirement for calculation of severity | Abundance measured before and after disturbance event | Inclusion | Containing two abundance measures in the 1-year periods immediately before and after the disturbance | To estimate the population growth rate when the disturbance event occurred |
| | Abundance measured under normal conditions | Inclusion | Containing four annual abundance transitions under normal conditions | To ensure the estimation accuracy of the variability of population growth rate under normal conditions |

References:
[1] Foster, D. R. Disturbance history, community organization and vegetation dynamics of the old-growth Pisgah Forest, south-western New Hampshire, USA. *J. Ecol.* **76**, 105–134 (1988).
[2] Turner, M. G. & Dale, V. H. Comparing large, infrequent disturbances: What have we learned? *Ecosystems* **1**, 493–496 (1998).
[3] Sæther, B. E. *et al.* Generation time and temporal scaling of bird population dynamics. *Nature* **436**, 99–102 (2005).

**Supplementary Table S4| Values of Akaike's information criterion (AIC) and Schwarz Bayesian Information Criterion (BIC) for the regression models**

The error distributions, link functions, the AIC values and the BIC values for the potential models of the generalized linear model.

| Error distribution | Link function | AIC | BIC |
|---|---|---|---|
| Gamma | Log | 161.76 | 167.50 |
| Gamma | Inverse | 163.27 | 169.00 |
| Gaussian | Identity | 287.36 | 293.10 |
